# A transgenic resource for conditional competitive inhibition of conserved *Drosophila* microRNAs

**DOI:** 10.1038/ncomms8279

**Published:** 2015-06-17

**Authors:** Tudor A. Fulga, Elizabeth M. McNeill, Richard Binari, Julia Yelick, Alexandra Blanche, Matthew Booker, Bruno R. Steinkraus, Michael Schnall-Levin, Yong Zhao, Todd DeLuca, Fernando Bejarano, Zhe Han, Eric C. Lai, Dennis P. Wall, Norbert Perrimon, David Van Vactor

**Affiliations:** 1 Department of Cell Biology, Harvard Medical School, Boston, Massachusetts 02115, USA; 2 Department of Genetics, Harvard Medical School, Boston, Massachusetts 02115, USA; 3 Howard Hughes Medical Institute, Harvard Medical School, Boston, Massachusetts 02115, USA; 4 Weatherall Institute of Molecular Medicine, Radcliffe Department of Medicine, University of Oxford, Oxford OX3 9DS, UK; 5 Department of Systems Biology, Harvard Medical School, Boston, Massachusetts 02115, USA; 6 Department of Developmental Biology, Sloan-Kettering Institute, New York City, New York 10065, USA; 7 Center for Cancer and Immunology Research, Children’s Research Institute, Children’s National Medical Center, 111 Michigan Avenue NW, Washington DC 20010, USA

## Abstract

Although the impact of microRNAs (miRNAs) in development and disease is well established, understanding the function of individual miRNAs remains challenging. Development of competitive inhibitor molecules such as miRNA sponges has allowed the community to address individual miRNA function *in vivo*. However, the application of these loss-of-function strategies has been limited. Here we offer a comprehensive library of 141 conditional miRNA sponges targeting well-conserved miRNAs in *Drosophila*. Ubiquitous miRNA sponge delivery and consequent systemic miRNA inhibition uncovers a relatively small number of miRNA families underlying viability and gross morphogenesis, with false discovery rates in the 4–8% range. In contrast, tissue-specific silencing of muscle-enriched miRNAs reveals a surprisingly large number of novel miRNA contributions to the maintenance of adult indirect flight muscle structure and function. A strong correlation between miRNA abundance and physiological relevance is not observed, underscoring the importance of unbiased screens when assessing the contributions of miRNAs to complex biological processes.

## Abstract (web summary)

Transgenic RNA ‘sponges’ that inhibit specific microRNAs are valuable tools for functional studies. Fulga *et al.* present a library of conditional microRNA sponges in *Drosophila*, and reveal widespread involvement of microRNAs in the maintenance of adult muscle structure and function.

## Graphical abstract

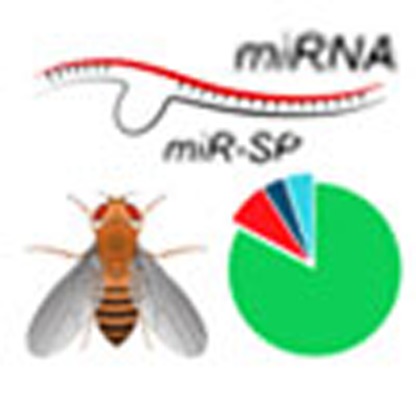

The last decade in biomedical sciences has brought renewed appreciation for the ancient world of RNAs and unanticipated dimensions of genome regulation by non-coding RNAs. In particular, microRNAs (miRNAs) have emerged as versatile rheostats of gene expression in development and disease. MiRNAs are ∼22 nucleotide endogenous non-coding RNAs that bind to specific miRNA recognition elements in target RNAs^1^,^2^. The overt consequence of miRNA activity is post-transcriptional silencing of gene expression primarily via RNA decay or translational inhibition^3^,^4^,^5^,^6^.

Despite rapid progress in understanding the molecular mechanisms underlying miRNA biogenesis and mechanisms of action, the biological functions of most miRNAs remain elusive at an organismal level. Aside from experiments in cell culture^7^,^8^, relatively little comprehensive screening has been performed *in vivo* to assess the functional complexity of the miRNA landscape^9^,^10^,^11^,^12^,^13^. This is partly due to a paucity of genome-wide resources for assessing miRNA loss of function (LOF). Null miRNA mutations obtained by targeted approaches will be invaluable for analysis of *in vivo* function^13^,^14^,^15^,^16^,^17^. However, comprehensive analyses of miRNA functions in specific tissues and in the dynamic context of the developing organism will also require precise spatiotemporal and gene dosage control. For this reason, we set out to develop a resource for conditional miRNA LOF that could enable unbiased screens for tissue-specific phenotypes.

The specificity of miRNA target recognition and binding is determined by Watson–Crick base pair complementarity. Recent studies suggest the existence of endogenous competitive inhibition regulatory systems that exploit this mechanism to control endogenous miRNA activity^18^,^19^,^20^,^21^,^22^,^23^,^24^. The same concept inspired the design of artificial competitive inhibitors that offer a powerful experimental approach for miRNA LOF studies. Such miRNA ‘sponge’ and ‘decoy’ technologies were successfully used to define a handful of miRNA functions in multiple species and biological contexts^25^. Mechanistically, this approach relies on the overexpression of transgenes encoding multiple copies of perfect complementary or ‘bulged’ miRNA target sites. Sponge (SP) transcripts sequester miRNAs, blocking access of target transcripts to endogenous target mRNAs, and thus creating a knockdown of miRNA activity that closely resembles hypomorphic or null mutants. When transgenically encoded, SPs can be deployed using binary modular expression systems, providing a versatile tool to study miRNA functions *in vivo* with spatial and temporal resolution^26^,^27^,^28^,^29^,^30^,^31^,^32^.

## Results

### A transgenic library of conditional miRNA competitive inhibitors

**Figure 1:**
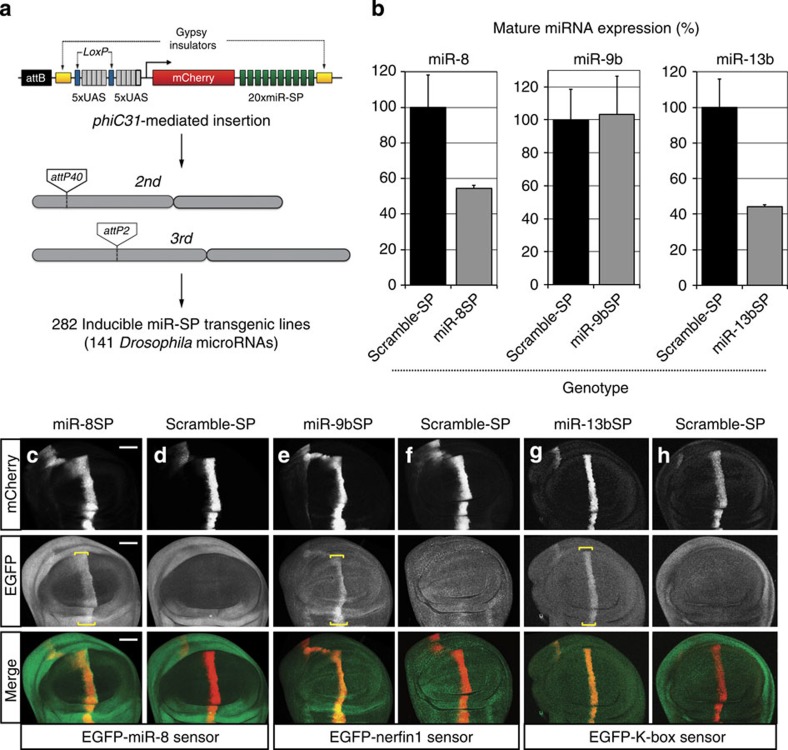
A transgenic library of conditional miRNA competitive inhibitors. (**a**) Second-generation SP elements consist of 20 miRNA binding sites with mismatches at positions 9–12 placed in the 3′-untranslated region of mCherry under the control of 10 tunable Gal4 UAS binding sites. The entire cassette was cloned in an *attB* vector containing gypsy insulators. *phiC31*-mediated positional integration was used to generate a library of 282 inducible lines covering 141 high-confidence *Drosophila* miRNAs, at defined landing sites on the second (attP40) and third (attP2) autosomes. (**b**) Quantification of endogenous miR-8, miR-9b and miR-13b mature miRNA levels using Taqman quantitative PCR in third instar larvae following ubiquitous expression (*tubulin-Gal4*) of corresponding miR-SP constructs compared with Scramble controls (Student’s *t*-test: 13b, *P*=0.02; error bars, s.e.m., *n*=3 biological replicates with 10 animals per sample). (**c**–**h**) Targeted expression of miR-8SP (**c**; scale bars, 50 μm), miR-9bSP (**e**) and miR-13bSP (**g**) with *ptc-Gal4* in wing imaginal discs ubiquitously expressing *tubulinEGFP-miR-8, tubulinEGFP-nerfin1 and tubulinEGFP-K-box* sensors respectively. Tissue-specific upregulation of sensor levels was observed in cells along the anterior–posterior boundary of the disc. No change was apparent following expression of a *Scramble-SP* control (**d**,**f**,**h**).

We have previously demonstrated that transgenic SP constructs can faithfully recapitulate known LOF phenotypes for several well-characterized miRNA genes^26^. Here we report the first transgenic library of conditional miRNA-SPs (miR-SPs), and describe several screens to detect novel miRNA functions required for adult viability, external morphology and flight muscle function in *Drosophila*. Second-generation (GenII) SP constructs were designed and cloned as recently described (Fig. 1a; see Methods section)^33^. A sliding window of 7–8 nucleotides encompassing linker and adjacent SP sequence was scanned to avoid cryptic overlap with existing *Drosophila* miRNA seed sequences in order to prevent off-target effects (Supplementary Data 1). For the purpose of this study, we focused on a subset of 141 high-confidence miRNAs^34^, 78 of which display ≥70% sequence similarity between *Drosophila* and humans^35^. Using the øC31 site-directed integrase system, we generated 282 transgenic lines carrying one miR-SP transgene on either the second or the third autosome, for each miRNA. Because we observed dose dependence when comparing expression of single and multiple SP insertions (see below), double transgenic lines were then created for each construct and used throughout this study. Analysis of endogenous miRNA levels following ubiquitous miR-SP^GenII^ expression in larvae (tubulin-Gal4 driver) indicated that the effect of miR-SP expression can vary depending on the miRNA. In some cases, we observed no effect on normal miRNA homeostasis (for example, miR-9b), in other cases a significant decrease in the abundance of mature target miRNAs was apparent (for example, miR-8 and miR-13b) (Fig. 1b). However, an *in vivo* miRNA reporter assay in wing imaginal discs revealed that a comparable decrease in miRNA activity is observed in all three cases (Fig. 1c–h).

### miRNA regulation of adult viability and external morphology

The importance of miRNA-dependent post-transcriptional regulation in animal development and disease is well documented in a large number of case studies. Surprisingly though, a comprehensive *in vivo* screen of 95 miRNA genes in *Caenorhabditis elegans* revealed that most individual miRNAs are dispensable or have limited impact on gross organismal development and innate adult behaviour^9^,^10^,^11^. To obtain an initial assessment of miRNA regulatory activities in *Drosophila*, we screened our attP2 and attP40 double-insertion miR-SP library with the ubiquitous tubulin-Gal4 driver, and assayed viability and gross morphological defects in eclosing adults. We also included in our screen two SP lines designed and characterized independently (for example, bantam^36^ and miR-1). Lines that displayed significant reduction in viability, defined by a stringent cutoff at a value equal or less than 1 s.d. of percent viability across the entire collection, were further validated in triplicate (see Methods section).

**Figure 2:**
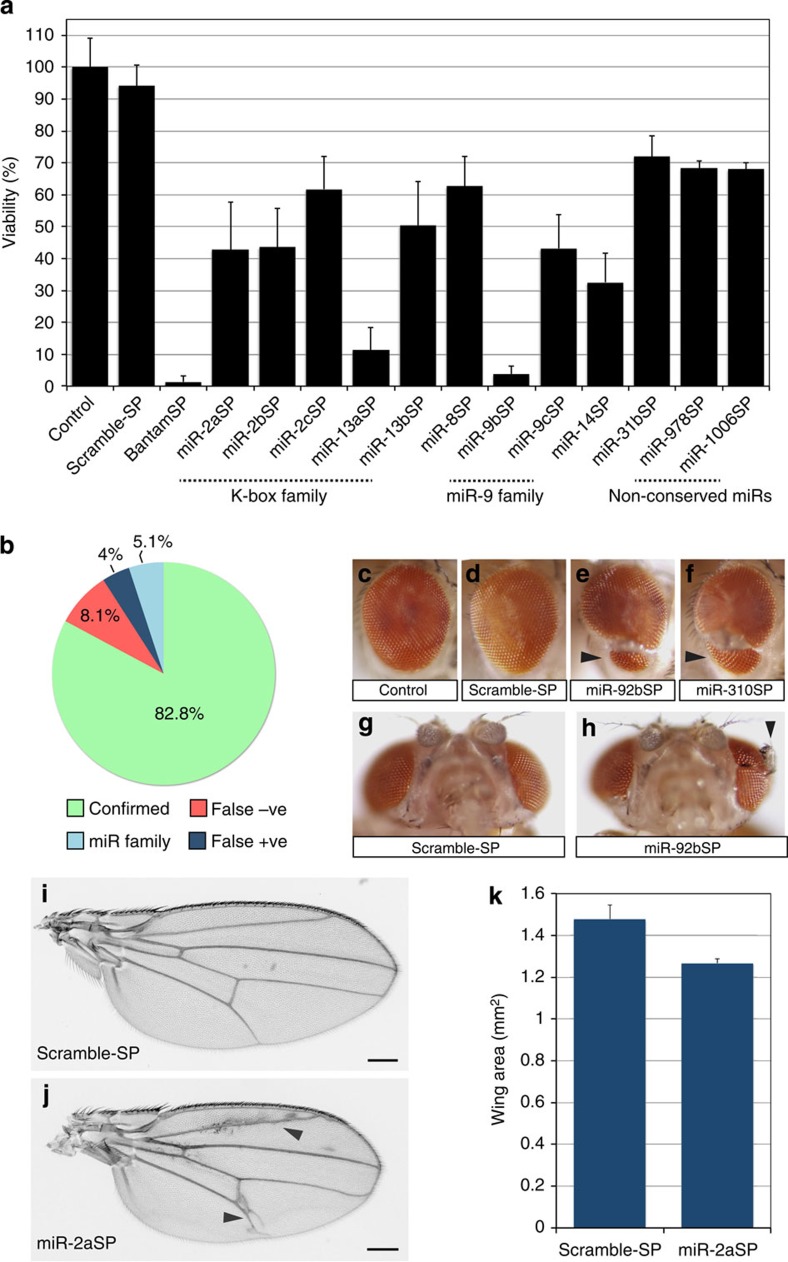
*Drosophila* miRNA phenotypes in viability and external morphology. (**a**) Viability defects following ubiquitous expression of SP library under the *tubulin-Gal4* driver. Data are displayed as average per cent viability relative to control; at least six independent replicate batches were analysed for each genotype (analysis of variance, *post hoc* analysis with Tukey–Kramer multiple comparisons test *P*≤0.01; error bars, s.d). (**b**) Benchmark comparison of viability phenotypes with *Drosophila* miRNA null mutants^13^. ‘Confirmed’ indicates same viability phenotype shared with SP and null. False negative (‘False –ve’) indicates miRNAs where null demonstrated viability impaired phenotypes, but miR-SP lines were viable. False positive (‘False +ve’) indicates miRNAs where a phenotype was observed with miR-SP but not in the null animal. ‘miR family’ represents miRNAs for which a similar seed sequence is shared and miR-SP lines display a viability phenotype that is only confirmed by an individual family member. The denominator for this chart was 99; we excluded all lines for which there was no null available, lines that were not tested by Chen *et al.*^13^, mutants removing entire clusters of multiple miRs, or mutants for which complementation was inconclusive (Supplementary Data 3). (**c–h**) Eye morphology defects (arrowheads) following inhibition of miR-92 activity. Genotypes: *+/tubulin-Gal4* (**c**), *+/Scramble-SP;tubulin-Gal4/Scramble-SP* (**d**,**g**), *+/miR-92bSP;tubulin-Gal4/miR-92bSP* (**e**,**h**) *+/miR-92bSP;tubulin-Gal4/miR-310SP* (**f**). (**i**–**k**) Expression of *miR-2aSP* results in wing vein patterning abnormalities (**j,** arrowheads) and an overall reduction in wing blade size (**j**; scale bar, 200 μm). Average wing size area was quantified in triplicate samples (*P*=0.004; error bars, s.e.m.) from *+/Scramble-SP;tubulin-Gal4/Scramble-SP* and *+/miR-2aSP;tubulin-Gal4/miR-2aSP* animals (**k**).

In total, 9% (13/143) of individual miR-SP transgenes rendered a statistically significant viability phenotype, ranging from lethal (0–5% viability) to semilethal (6–50% viability) to subviable (50–70% viability; Fig. 2a). Some lines displayed penetrance below our stringent cutoff that may reflect partial LOF in essential miRNA functions (Supplementary Data 2). In principle, some SPs should be able to inhibit multiple miRNAs in a conserved family. Supporting this argument, several hits in the viability screen belonged to the K-box family (miR-2a, miR-2b and miR-2c, and miR-13a and miR-13b) and the miR-9 family (miR-9b and miR-9c). Previous analysis of K-box miRNA double mutants revealed functional redundancy for lethality^37^. We tested several of our hits using a complementation assay where the lethal phenotype of a single SP insertion was compared with the same SP carried over a deficiency (Df/+) at the endogenous locus (as described in ref. 26). By this classical criterion, miR-2aSP, miR-2bSP and miR-8SP displayed increased penetrance, and thus non-complementing behaviour, over Df (Supplementary Fig. 1a). Interestingly, among miR-9 family members tested (miR-9bSP and miR-9cSP), only miR-9cSP was strongly uncovered by Df (Supplementary Fig. 1a), suggesting some degree of specialization for endogenous miRNA functions within the conserved family.

While our manuscript was under review, a screen of miRNA deletion mutants for lethal phenotypes was published^13^, thus allowing a broad benchmark comparison of miR-SPs with independent viability data (summary in Fig. 2b). Of the miRNAs we tested for viability (141 miR-SP^GenII^ strains plus two other constructs; Supplementary Data 2), null alleles exist for 115. Sixteen of these mutants were deemed as not comparable as benchmarks because they either (a) remove multiple clustered miRNA genes, (b) fail to display non-complementation over large Dfs at each locus (that is, not genetically validated) or (c) they were not tested for lethality by Chen and colleagues^13^ (Supplementary Data 3). In addition, 27 miR-SP^GenII^ constructs correspond to miRNAs for which no null allele currently exists (Supplementary Data 3). Thus, we compared adult viability phenotypes of null and tubulin-Gal4;UAS-miR-SP for 99 genes (Fig. 2b and Supplementary Data 3; ref. 13). The vast majority of the viability phenotypes in our screen match the published data (82.8%; green in Fig. 2b and Supplementary Data 3). Several miR-SPs did show viability defects that were not observed in corresponding nulls; however, several were members of highly conserved families likely to display functional redundancy as previously observed for K-box miRNAs (light blue in Fig. 2b and Supplementary Data 3; ref. 37), thus leaving 4% as conclusive false positives (miR-14, miR-79, miR-307 and miR-975; dark blue in Fig. 2b and Supplementary Data 3). Finally, some null mutants displayed lethality that was not detected in our tubulin-Gal4;UAS-miR-SP screen, as expected in screens of hypomorphic mutants (for example, using RNA interference or chemical mutagenesis). Overall, the false-negative rate for viability was 8.1% (red in Fig. 2b and Supplementary Data 3).

We expected that SP activity would be dose dependent relative to endogenous levels of targeted miRNA, thus allowing us to control the strength of conditional inhibition. To test this, we compared the viability of 1 × and 2 × SP insertions with tubulin-Gal4 for several of the hits in our screen, including miR-2bSP, miR-8SP, miR-9bSP and miR-9cSP. In each case, the 2 × SP gave a more penetrant adult lethal phenotype than 1 × SP (Supplementary Fig. 1b). In addition, it is likely that intrinsic differences in miR-SP architecture can influence their efficacy. For example, a previous study using a different lethality assay and SP design reported viability defects following miR-92 competitive inhibition^28^. However, our individual strains with miR-SP^GenII^ constructs directed against miR-92 family members (miR-92a,b and miR-310/311/312/313) did not display significant lethality, despite the fact that other phenotypes can be detected with SPs directed against this family (see below).

Examining external morphology, we have previously reported that miR-SPs can replicate the deformed adult leg phenotype caused by loss of miR-8 function^26^,^38^. This was confirmed with our GenII miR-8SP lines (Supplementary Fig. 2a–c). GenII SP strains also recapitulated the miR-9-dependent notching of the posterior wing blade margin^39^,^40^,^41^ (Supplementary Fig. 2d–f). In the adult compound eye, we also observed a novel and highly penetrant morphological phenotype using miR-92bSP, characterized by an apparent invasion of the head cuticle into the retina (Fig. 2c–e) or even more marked ectopic outgrowth within the retinal field (Fig. 2g,h). The identical phenotype was observed for miR-310SP, another member of the miR-92 family (Fig. 2f), suggesting some degree of functional redundancy between endogenous members of this miRNA family. In addition, we found that surviving miR-2aSP adults displayed a novel vein patterning defect and a decreased wing size (Fig. 2i–k). However, the overall frequency of gross morphological phenotypes was quite low (0.7–1.4%, depending on phenotype).

### Tissue-specific miRNA function in Drosophila muscle

**Figure 3:**
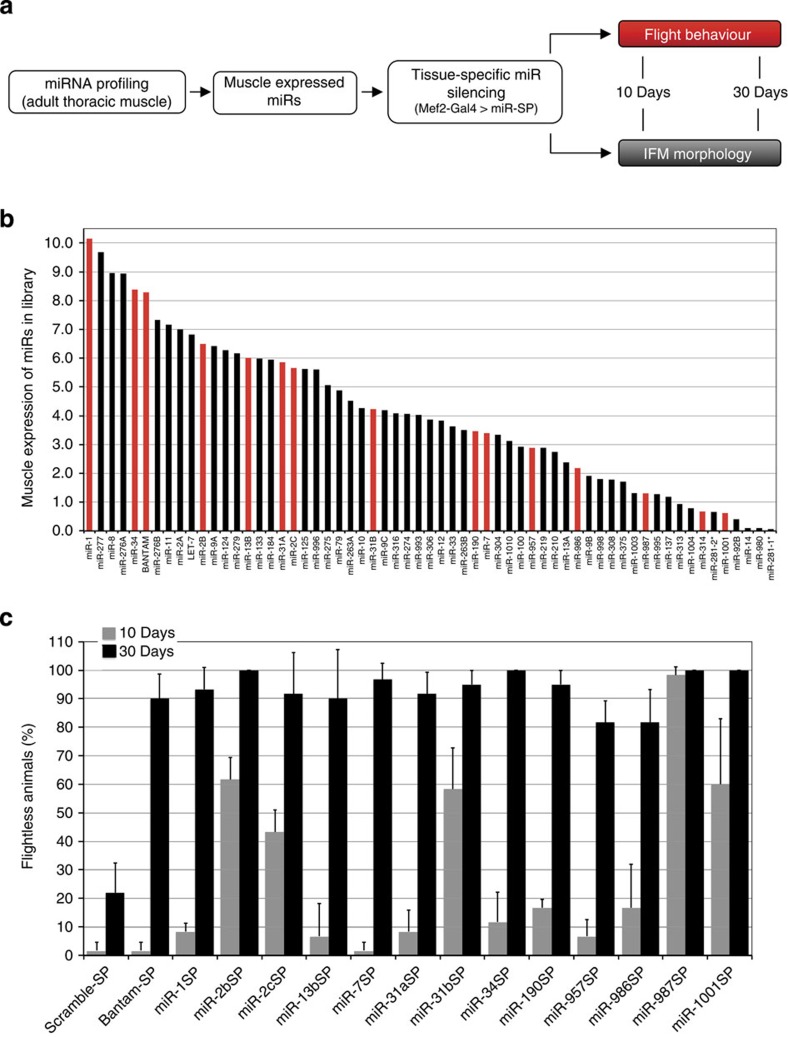
Tissue-specific *in vivo* screen for miRNAs regulating muscle function and maintenance. (**a**) Screen strategy diagram. Muscle-expressed miRNAs were profiled in the adult thoracic muscle tissue, and silenced by driving corresponding miR-SPs with the *dMef2-Gal4* driver. Flight behaviour and IFM morphology was assessed at 10- and 30-day-old animals. (**b**) miRNA microarray profiling of the thoracic muscle tissue. For simplicity, only miRNAs with detectable expression are shown. Red bars denote positive hits in the primary muscle screen. (**c**) Positive hits from the flight screen. ‘Flightless phenotype’ was defined at a value above twice the s.d. of *Scramble-SP* controls in 30-day-old animals (analysis of variance, *post hoc* analysis with Tukey–Kramer multiple comparisons test *P*≤0.001; error bars, s.d., *n*=3 replicates of 20 animals). Flight behaviour is shown for 10-day-old animals in grey bars and for 30-day-old animals in black bars. *indicates less abundant strand from the mentioned hairpin structure.

Although a growing body of evidence suggests that miRNAs play vital roles in maintaining the integrity and function of adult tissues^42^,^43^,^44^, comprehensive interrogation of such phenotypes has been challenging. To realize the potential of our SP library for unbiased discovery of tissue-specific miRNA functions, we next sought to screen for miRNAs that regulate adult muscle morphology, maintenance and function. We first determined the muscle expression of the miRNAs present in our collection. Total RNA was isolated from dissected adult thoracic muscles, and relative expression levels were determined using a miRNA microarray platform (Fig. 3a). *Drosophila* miRNA array signals were obtained by fitting a linear model to the log2-transformed probe intensities. This analysis detected 61 miRNAs expressed across a broad range of relative levels in adult thoracic muscles (Fig. 3b; miR-SP^GenII^ strains were available to test 58 of these).

To disrupt the activity of these candidate miRNAs selectively in the muscle tissue from embryonic stages through adulthood, 2 × miR-SP constructs were expressed using a *dMef2-Gal4* driver. Flight behaviour and indirect flight muscle (IFM) morphology were assessed in adult progeny at 10 and 30 days post eclosion (Fig. 3a). Analysis of 30-day-old animals revealed that 14 miR-SP lines rendered a penetrant ‘flightless’ phenotype (black bars in Fig. 3c). These included miR-SPs targeting bantam, miR-1, the K-box family (miR-2b, miR-2c and miR-13b displayed strong phenotypes; miR-2a and miR-13a were flight impaired but fell below our stringent cutoff; Supplementary Data 4), miR-7, the miR-31 family, miR-34, miR-190, miR-957, miR-986, miR-987 and miR-1001. All but one of these lines (miR-987SP) appeared normal or displayed mildly impaired flight behaviour at 10 days (grey bars in Fig. 3c). However, when we then assayed miR-987SP adults at 4 days post eclosion, we found normal flight behaviour relative to control (1.7±2.9% non-fliers in miR-987SP compared with 0% in Scramble-SP). Therefore, all behavioural phenotypes recovered in our muscle screen displayed a progressive, age-dependent loss of flight. These miRNA genes were also evenly distributed across the range of expression levels (Fig. 3b red bars), showing little correlation with endogenous miRNA abundance.

**Figure 4:**
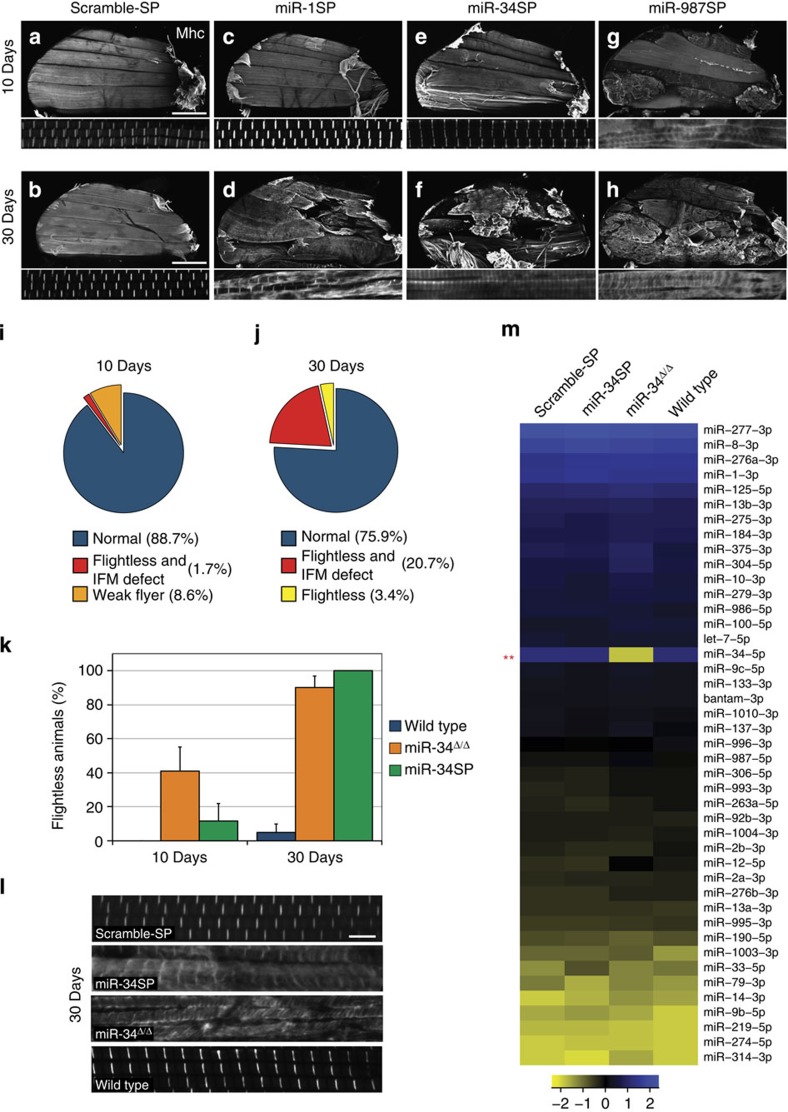
Twelve miRNAs are required to maintain flight muscle structure. Fifty-eight lines were assayed for flight at 10 and 30 days post eclosion, and all lines that displayed significant flight deficits were then assayed for IFM morphology (**a**–**h**). Sagittal bisections of the adult thorax stained for actin and myosin heavy chain (Mhc) shown at low (top panel) and high magnification (bottom panel). Normal IFM and sarcomere morphology in 10- and 30-day-old Scramble-SP controls (**a**,**b**; scale bars, 200 μm), late-onset IFM phenotype following miR-1SP expression (**c**,**d**) or miR-34SP expression (**e**,**f**), and early-onset IFM defects in *miR-987SP* animals (**g**,**h**). A summary of the lines that display flight and IFM phenotypes at 10 days post eclosion (**i**) is shown for comparison with the 30-day results shown in **j**; red represents all SP lines that display both flight and IFM defects, whereas orange and yellow represent animals with no detectable IFM morphology defect that were flight impaired or flightless, respectively. (**k–l**) Comparison of *miR-34SP* and *miR-34*^*Δ*/*Δ*^ null mutants. Null mutant adults (orange bars) display a stronger flightless phenotype at 10 days but are comparable to *miR-34SP* (green bars) at 30 days (**k**); error bars, s.e.m., *n*=3 replicates of 20 animals. IFM sarcomere morphology and Mhc distribution and pattern are comparable in *miR-34SP* and *miR-34*^*Δ*/*Δ*^ null mutants at 30 days (displaying 15.7% penetrance (*n*=19), compared with 25% in *miR-34SP*; **l**; scale bar,5 μm). (**m**) NanoString nCounter profiling of adult thoracic muscle. All miRNAs expressed above background values are represented. Only the levels of mature miR-34–5p were substantially reduced in the null mutant. Statistical significance was established in this case by comparing the expression values of *miR-34*^*Δ*/*Δ*^ to the wild-type control using the NanoStringNorm package in R (*t*-test, **P<0.003). For all other genotypes, statistical significance was established by comparing the miR-SP values, against *miR-34*^*Δ*/*Δ*^, *Scramble-SP* and wild-type controls. No other endogenous miRNA levels change significantly in *miR-34SP* or *miR-34*^*Δ*/*Δ*^ animals compared with Scramble-SP or wild-type controls (see Methods section).

Adult flight behaviour is primarily dependent on the activity of the IFMs. To assess the impact of miRNA inhibition on muscle morphology, IFM myofibril structure was examined in sagittal bisections of the thorax stained for F-actin and myosin heavy chain. At 30 days, 12 of the 14 flightless SP lines showed marked defects in IFM muscle integrity and sarcomere organization (Fig. 4a–h; Supplementary Fig. 3), with a relatively broad range of penetrance (Supplementary Fig. 4). Only miR-7SP and miR-13bSP showed no detectable IFM abnormalities at this level of resolution (yellow wedge in Fig. 4j). Notably, miR-987SP animals displayed a detectable defect in gross IFM or myofibril morphology at 10 days (red wedge in Fig. 4i). Thus, despite strong *dMef2-Gal4*-dependent expression starting in the mesoderm at embryonic stage 7, our SP screen detected many age-dependent IFM phenotypes, but no obvious defects in muscle development.

Among the candidate miRNAs identified in our screen, miR-1 is considered an ‘archetypal’ muscle miRNA whose sequence and expression pattern appears to be evolutionarily conserved from flies to mammals. In *Drosophila*, miR-1 null mutations display paralysis, severe disruption of somatic muscle tissues and early larval death, preventing analysis of function during adult life^45^,^46^,^47^. Examination of 30-day-old escapers expressing miR-1SP uncovered a highly penetrant flightless phenotype and severe degeneration of IFM muscle fibres (Fig. 3c and 4d). These results highlight the capacity of miRNA SPs to complement studies where complete LOF renders early developmental lethality.

Unlike miR-1, miR-34 had not previously been analysed in *Drosophila* muscle despite mounting evidence implicating this conserved miRNA in muscle function (Supplementary Table 1). Thus, we sought to confirm this function for miR-34 by examining a null mutation^42^. Indeed, homozygous null animals (*miR-34*^*Δ*/*Δ*^) display age-dependent deficits in flight behaviour that are slightly more severe than miR-34SP at 10 days but reach comparable levels at 30 days (Fig. 4k). Moreover, IFM morphology comparisons confirm that *miR-34*^*Δ*/*Δ*^ nulls display the same abnormal morphology and distribution of myosin heavy chain characteristic of *dMef2-Gal4;miR-34SP* animals at comparable penetrance (Fig. 4l). Because the miR-34 muscle phenotype was qualitatively similar to many of the other hits in our flight screen, we wanted to confirm that *miR-34*^*Δ*/*Δ*^ and miR-34SP did not cause altered expression of other muscle-expressed miRNAs required for muscle maintenance. Thus, we used sensitive NanoString *nCounter* profiling to monitor miRNAs levels in the adult thorax (see Methods section). Aside from the loss of miR-34–5p in null mutants, no other miRNAs were significantly changed compared with controls (Fig. 4m and Supplementary Data 5), suggesting that miR-34 acts independently of other conserved miRNAs in this context.

## Discussion

In this study, we describe a transgenic *Drosophila* resource for conditional competitive inhibition for 141 high-confidence miRNAs, as a versatile toolkit for discovery and tissue-specific analysis of miRNA functions *in vivo*. This resource is highly complementary to collections of miRNA gene deletions that offer chronic, complete and systemic LOF^48^,^13^. Similar to the chemical mutagenesis and RNAi methods typically used to detect novel loci in genome-wide functional screens^49^, miR-SPs usually produce partial LOF; however, this feature combined with the spatial–temporal specificity conferred by the huge arsenal of Gal4 drivers (for example, http://flystocks.bio.indiana.edu/) empowers the miR-SP approach with many advantages for analysis of post-embryonic and cell- or tissue-specific functions.

Overall, the occurrence of significant adult viability and external morphology defects following ubiquitous miRNA inhibition in *Drosophila* appears to be comparable to the frequency of phenotypes resulting from systemic loss of miRNA function in *C. elegans*^10^. The relatively low frequency of external morphology defects (3.5% overall; *n*=5/143; Fig. 2 and Supplementary Fig. 2) and the low false discovery rates observed in our tubulin-Gal4 screens (Fig. 2b), suggest that transgenic SPs are largely free from significant off-target effects. Interestingly, our novel tissue-specific screen identified a much greater percentage of miRNAs required for the form and function of adult flight muscle (24%; *n*=14/58; Fig. 4h). Our analysis suggests that disruption of miR-34 and 11 other miRNAs can induce a progressive disruption of IFM structure and function, thus uncovering a substantial regulatory landscape for muscle maintenance and/or homeostasis.

Recent studies suggest that vertebrate orthologues for several of the conserved miRNAs required for muscle maintenance in our screen (miR-1, miR-7, miR-31 and miR-34, and the K-box orthologue miR-23) are associated with muscle physiology in vertebrate species (Supplementary Table 1). However, to our knowledge, only miR-1 and miR-34 have been implicated by LOF in vertebrate cardiac and/or skeletal muscle function^43^,^50^. Interestingly, loss of *Drosophila* miR-34 has been reported to induce late-onset brain degeneration^42^, raising the intriguing possibility of a general tissue maintenance theme. Of course, future study is needed to distinguish between events that may trigger active degenerative processes versus those that disrupt ongoing replenishment of protein networks in muscle. It may also be interesting to test these muscle-maintenance miRNAs for degenerative phenotypes in other tissues. Although future comparisons with null mutations will be required to validate many of these novel loci, the fact that most of these miRNAs were not previously known to support muscle maintenance highlights the potential of the miR-SP library for tissue-specific screening. In conclusion, the library of transgenic SPs reported here represents a valuable resource for unbiased and conditional LOF screens in the intact organism.

## Methods

### Genetics and miR-SP library generation

*Drosophila stocks*. The following Gal4 drivers were obtained from the Bloomington Stock Center and crossed with miR-SP lines to drive ubiquitous, wing disc and mesodermal expression: *tubulin-Gal4, patched (ptc)-Gal4* and *dMef2-Gal4*, respectively. The transgenic lines containing 3′-untranslated region sensors for miR-8, nerfin, and K-box miRNAs, were previously described^51^,^52^,^53^. miR-1SP construct was generated in the Han laboratory by introducing 10 repetitive miRNA complementary sequences (5′-GGTACGTTTAGCGTAAGTTAT-3′ synthesized by GenScript) separated by four nucleotide linkers 5′-CGCG-3′ into the pUAST vector. Bantam-SP was a generous gift from Steve Cohen.

*Conditional miR-SP collection*. miR-SP constructs were designed with a silencing cassette of 20 repetitive miRNA complementary sequences separated by variable four-nucleotide linker sequences, and assembled as previously described^26^. To avoid off-target effects, the combined miRNA and linker sequences were checked against every mature miRNA sequence in the *Drosophila* genome. The entire cassette was then cloned into the 3′-untranslated region of mCherry between NotI and XbaI in a modified pWALIUM10-moe vector (ref. 54, http://www.flyrnai.org/TRiP-HOME.html) carrying the white+ selectable marker and flanking insulator sequences (as described in ref. 33) To obtain miR-SPs with relatively equal expression and avoid epigenetic positional effects, transgenic flies were generated using phiC31 site-specific genomic integration in specific landing sites on the second (*attP40*) and third (*attP2*) *Drosophila* autosomes (Genetic Services Inc.). Both attP2 and attP40 insertion site stocks are viable as homozygotes and have been characterized by the Perrimon laboratory as controls for genetic analysis of muscle ageing and viability phenotypes that run out to 56 days^55^; attP40 insertions are carried as heterozygotes in all SP screens carried out. Insertion of Scramble-SP sequence at the attP2 and attP40 sites acted as control. The sequences of all designed miR-SP constructs are listed in Supplementary Data 1.

*Mature miRNA quantification*. Total RNA was isolated according to the miRVana miRNA kit protocol without enrichment for miRNAs (Invitrogen) from ubiquitously expressing miR-SP or Scramble wandering third instar larvae with intestines removed. Real-time quantitative PCR was performed using a standard TaqMan MicroRNA assay kit protocol on an Applied Biosystems 7900HT Sequence Detection System (Applied Biosystems). Reaction volumes, cycles and analysis were performed as described^56^ with the exception that expression values are expressed relative to S2 rRNA expression.

*Immunostaining of imaginal discs*. Larvae were dissected in ice-cold PBS. Discs were fixed in 4% paraformaldehyde (PFA) at room temperature for 20 min, washed in PBS and PBST (0.01% Triton X-100), blocked with 5% normal goat serum in PBST and incubated with primary antibody anti-GFP (Molecular Probes A6455, 1:500) overnight at 4 °C, washed three times in PBST and then incubated with secondary Alexa Fluor 488 goat anti-rabbit IgG (Molecular Probes A-11008, 1:2,000) for 3 h at room temperature. Discs were then washed, mounted in SlowFade Gold antifade reagent (Invitrogen) and imaged with a × 20 objective on a Nikon A1R confocal.

### Ubiquitous miR-SP expression and analysis

Crosses to examine lethality were carried out at 27 °C with 12 males carrying the miR-SP on the second and third chromosome, and 25 *tubulin-Gal4/TM3* virgins allowed to mate for 1 day in a vial, then transferred to a bottle on the second day and finally transferred to a second bottle on day 4. After 2 days, the adult flies in the final bottle were discarded. Eclosed animals were collected every day up to 6 days after first eclosion and promptly counted. Flies were scored for and against mCherry expression, or against the *TM3* balancer. To account for subtle contribution(s) of TM3 to viability phenotypes, balanced drivers were crossed to *Canton-S* to establish a correction factor. Raw data are shown in Supplementary Data 2. miR-SP lines displaying lethality in the above assay were crossed again in vials with six miR-SP males and 10 *tubulin-Gal4* virgins. Each genotype was set up in triplicate. Crosses were flipped every 2 days into a new vial for 5 days, after which eclosed animals were collected and counted as described above. All lines were screened as double-insert (2 × ) stocks to increase phenotypic penetrance because comparisons between 1 × for several miR-SPs showed consistent dose dependence (Supplementary Fig. 1b).

Gross morphology of ubiquitously miR-SP expressing animals was examined with specific attention to retina (size/shape/pattern/pigmentation/bristle), wing (size/shape/veination/bristle and hair pattern), leg (length/shape/segment morphology/bristle pattern) and body (size/shape/bristle pattern). For the analysis of leg and wing morphology, cuticle preparations were prepared by dehydrating in a series of ethanol dilutions. Muscle was then cleared in xylene and tissues were mounted in Cytoseal-60 (Cole-Parmer). Wings and legs were imaged with Nikon Digital Sight DS-Fi1 colour camera on a Nikon 80i upright microscope at × 4. A 2 × 2 montage was taken and stitched together using NIS-Elements software. Area was calculated from stitched images using Fiji image processing package ( http://fiji.sc/Fiji).

### Muscle-specific miR-SP expression and analysis

Crosses to examine adult flight behaviour were carried out as described above (lethality assay) with the exception that *dMef2-Gal4* virgins were used to drive muscle-specific expression of the miR-SP. Eclosed animals were collected every day up to 6 days after first eclosion, and then aged for 10 or 30 days. Animals were flipped to new food vials every other day to maintain integrity of the collection. Flight assay was carried out at ambient temperature in a dark room in an illuminated arena with the following dimensions: *H*=65 cm, *W*=64 cm and *D*=51 cm. Flies were sorted into three groups of 20 for each gender, a minimum of 1 h before the assay. After 1 h recovery from brief anaesthesia, we found that no wild-type control flies (Canton-S; *dMef2-Gal4* raised at 27 °C to elevate Gal4 activity) hit the target area at 10 days of age. A small number of control animals fly poorly, ending up in the outer ring, when the flies are reared and aged to 30 days at 27 °C. This result was consistent with observations following 24 h of recovery from CO_2_. All tests were completed at the same time of day. Animals were flipped into a vial with no food directly preceding the assay. Flies were dropped through a funnel from a height of 74 cm centred above three concentric circles (diameter: 7 cm inner circle, 15 cm middle circle and 21 cm outer circle), and the number of animals in each circle was scored from an image of the arena taken immediately after landing. Flies falling within the first two circles were counted as ‘non-fliers’ (raw data in Supplementary Data 4). A threshold for ‘non-fliers’ was set at 2 s.d.’s of *Scramble-SP*, followed by analysis of variance and Tukey–Kramer multiple comparisons test to assess significance for each SP line above threshold.

IFM morphology was assessed only in flightless animals and controls. Wings, legs and abdomen removed from adult flies and the thorax muscles were soaked in relaxing solution (20 mM phosphate buffer, (pH 7.0), 5 mM MgCl_2_ and 5 mM EGTA) for 5 min. Thoraces were then moved to 4% PFA in relaxing solution for 10 min and then transferred to a 5% agarose gel plate covered with PBT (PBS+0.2% Triton X-100). Thoraces were then bisected sagittally with a scalpel blade (Fine Science Tools) and blocked in relaxing solution+3% heat inactivated goat serum for 20 min before fixation in 4% PFA in PBT for 10 min. Immunohistochemistry was carried out on the hemi-thoraces with mouse α-MHC (myosin heavy chain) (1:50 in PBT; ref. 57) followed with incubation in goat α-mouse 568 (1:200 in PBT, Invitrogen A-11031) and Alexa Fluor phalloidin 488 (1:500 in PBT, Invitrogen A12379). Samples were mounted in SlowFade Gold antifade reagent (Invitrogen). Hemi-thoraces were imaged using a Nikon Ti-E and A1R confocal with × 10 and × 100 objectives using NIS-Elements acquisition software. A minimum of *n*=4 hemi-thoraces were imaged for each genotype to account for variable expressivity (Supplementary Fig. 4); samples with evidence of tissue damage due to improper dissection were excluded before analysis of the results. Max-intensity projections were obtained using the NIS-Elements analysis software.

*Profiling miRNA muscle expression*. Muscle tissue was dissected from the thorax of adult flies and isolated using the standard Trizol (Invitrogen) protocol followed by RNeasy Plus kit (Qiagen) clean up with DNase treatment. RNA was labelled with Cy5 following Agilent standard protocol. Agilent microarrays covering 152 *Drosophila* miRNAs were designed as previously described^58^, with miRNA probes of varied lengths to equalize melting temperatures to 55 °C. MiRNA expression data were analysed using the AgiMicroRNA Bioconductor Package version 2.0.1 (ref. 59). The software is implemented in the open-source statistical scripting language R and is integrated into the Bioconductor project ( http://www.bioconductor.org) under the general public licence (GPL) licence. For data pre-processing, a target file was generated to assign each scanned data file to the appropriate experimental group. Scanned data from the Agilent Feature Extraction image analysis software were imported into an R object that stores the relevant probe and raw intensity data information needed for the pre-processing. Raw array data were normalized using quantile normalization, and we obtained the miRNA gene signal by fitting a linear model to the log2-transformed probe intensities. This model produced an estimate of the miRNA gene signal corrected for probe effects. To evaluate differences in the individual gene expression between experimental groups, the absolute value of the difference in total expression was computed for each of the miRNAs sampled on the array.

*NanoString nCounter miRNA profiling*. All crossed were carried out at 27 °C. Thoraces from 1- to 2-day-old adult females of relevant genotypes (*dMef2-Gal4>miR34b*; *dMef2-Gal4>Scramble-SP*; *miR-34*^*Δ*/*Δ*^ (a gift from Nancy Bonini) and *Iso white-1,2,3*) were dissected (*n*≥4) in PBS in biological duplicates. Total RNA was extracted using the miRNeasy kit (Qiagen). Purified RNA was concentrated using Amicon Ultra-0.6 Centrifugal Filters (Millipore). For each sample, ∼100 ng total RNA was loaded into the nCounter *Drosophila* miRNA Assay (NanoString) and processed according to the manufacturer’s protocols. Briefly, miRNAs were ligated, hybridized to reporter probes at 65 °C for 12 h and prepared on the nCounter Prep Station before being digitally counted at 555 Field of view (FOV) on the nCounter Digital Analyzer. The raw data counts were analysed using the NanoStringNorm R package^60^. The data were normalized using the geometric mean of the six positive controls, and then it was background corrected by subtracting the mean and 2 s.d.’s of the six negative controls. Finally, the data were normalized for sample/RNA content using the geometric mean of three housekeeping genes. Normalized miRNA expression levels were log2 transformed and analysed using a *t*-test to identify differentially expressed miRNA between samples. Heatmaps were generated using the gplots R package with the log2-transformed and normalized values of the experiment. Subsequently, for each condition, the mean of the two replicates was taken and the data were centred and scaled by subtracting for each condition the mean values and dividing it by the s.d.

## Additional information

**How to cite this article**: Fulga, T. A. *et al.* A transgenic resource for conditional competitive inhibition of conserved *Drosophila* microRNAs. *Nat. Commun.* 6:7279 doi: 10.1038/ncomms8279 (2015).

## Supplementary Material

Supplementary Information Supplementary Figures 1-4, Supplementary Table 1 and Supplementary References.

Supplementary Data 1 Sequences of miRNA SP constructs designed for the collection. Four nucleotide variable linkers separating each SP repeat are shown in red bold font and restriction sites used for cloning are in blue.

Supplementary Data 2 Primary lethality screen: ubiquitous miR-SP expression (*tubulin-Gal4* driver). Viability was defined as percentage of eclosing adults from +/*miR-SP;tubulin-Gal4/miR-SP* animals relative to +/*miR-SP;TM3,Sb/miR-SP* controls from the same genetic cross. Note that reduced viability in *miR-79SP*, *miR-307SP*, *miR-314SP* and *miR-975SP* crosses are likely to be off target effects, and these lines were subsequently removed from the collection.

Supplementary Data 3 Benchmark comparison of viability phenotypes to miRNA null mutants.

Supplementary Data 4 Primary flight screen: muscle-specific miR-SP expression (*dMef2-Gal4* driver). Each genotype was tested in triplicate with n=20 animals for each experiment, 10 days and 30 days after eclosion.

Supplementary Data 5 The raw data for Nanostring nCounter profile of mature steady state miRNA levels in *miR-34* null and *dMef2-Gal4;miR-34SP* compared to wild type and *dMef2- Gal4;UAS-Scramble-SP* controls, respectively. Data was analyzed using the NanoStringNorm R package; p values are indicated for each pairwise comparison of expression values (t-test).
