# Supplementary material for: A transgenic resource for conditional competitive inhibition of conserved Drosophila microRNAs
Source: Nat Commun. 2015 Jun 17;6:7279. doi: 10.1038/ncomms8279 (PMC4471878; doi:10.1038/ncomms8279)
Supplement: Supplementary Information — Supplementary Figures 1-4, Supplementary Table 1 and Supplementary References. [file ncomms8279-s1.pdf]

Supplementary Figures

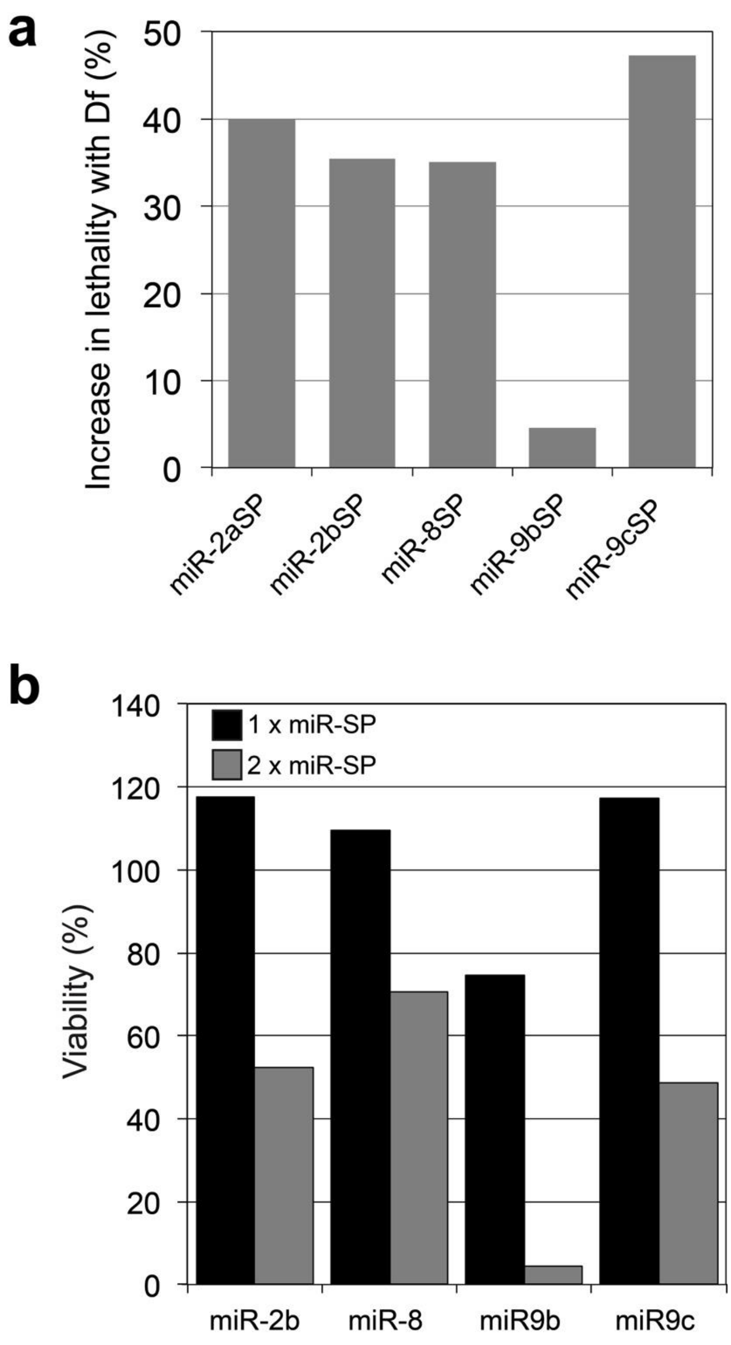

Supplementary Figure 1: (a), Complementation assays were performed to determine if lethality induced by single insert expression (*tubulin-Gal4;1x miR-SP*) is enhanced by a deletion (Df) at the corresponding endogenous locus; *miR-2bSP*, *miR-8SP* and *miR-9cSP* show significant increased percentage (%) lethality with Df. **(b)**, Using adult viability as a basis of phenotypic comparison, outcomes from crosses of *tubulin-Gal4* to single insert (1x) or double insert (2x) SP lines are shown for four of the hits in the screen. All of these miR-SPs display dose-dependence.

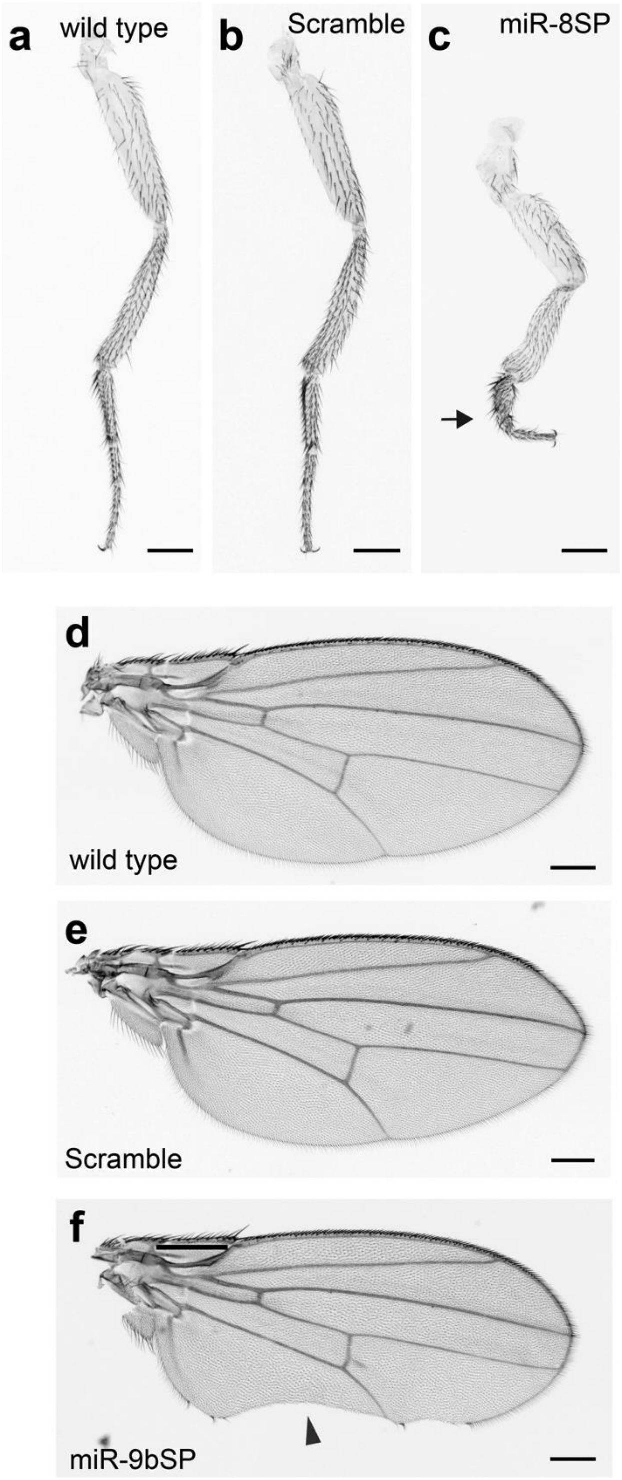

Supplementary Figure 2: Second generation miR-SP constructs recapitulate previously characterized miRNA LOF morphological phenotypes. **(a-c)** Adult third leg morphology from wild type control **(a)**, *+/Scramble-SP;tubulin-Gal4/Scramble-SP* **(b)**, and *+/miR-8SP;tubulin-Gal4/miR-8SP* **(c)** animals*.* **(d-f)** Cuticle preparations of wings from wild type control **(d)**, *+/Scramble-SP;tubulin-Gal4/Scramble-SP* **(e)**, and *+/miR-9bSP;tubulin-Gal4/miR-9bSP* **(f)** animals, Scale bars are 200µm*.*

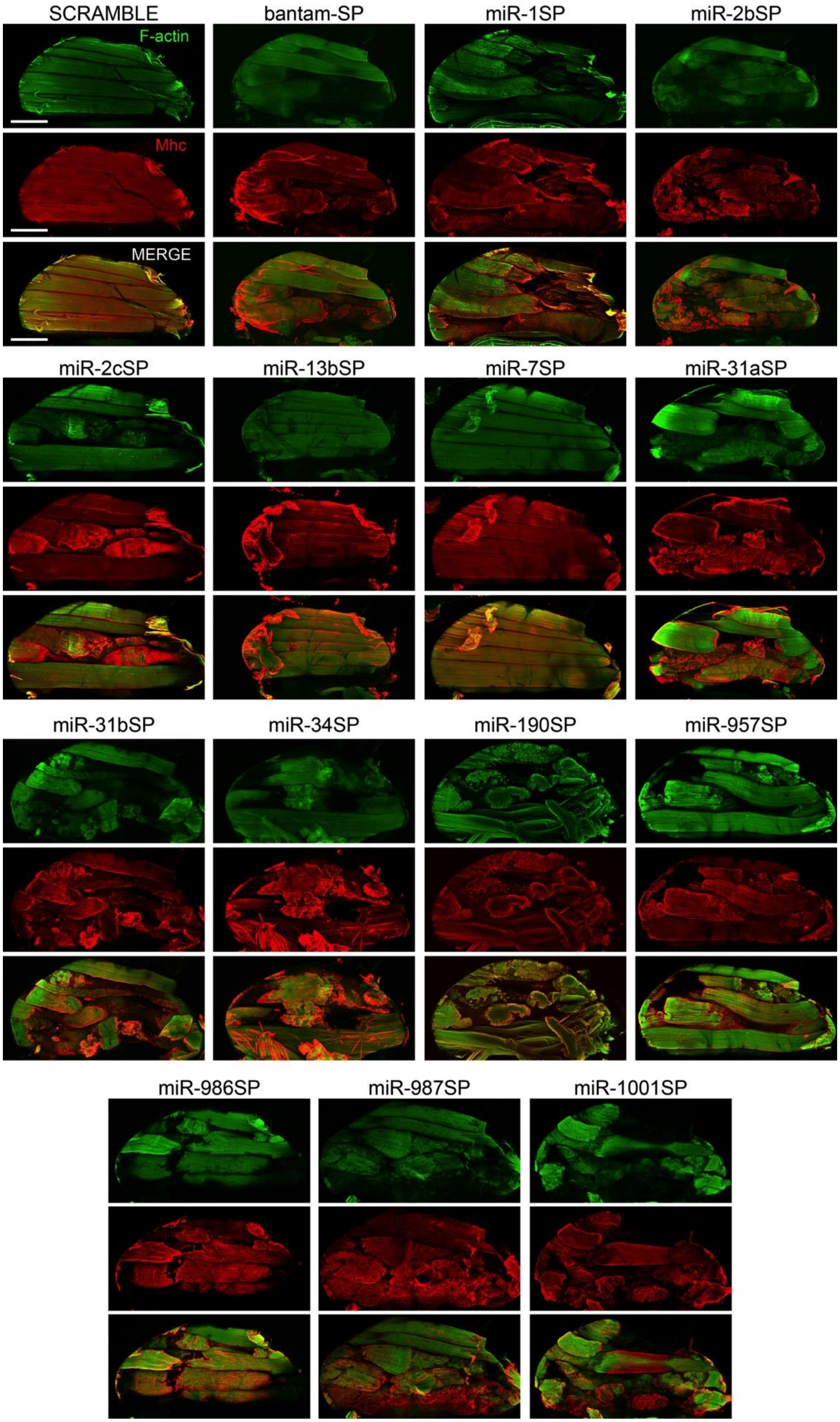

Supplementary Figure 3: IFM morphology phenotypes of all positive hits from the primary flight screen. Adult indirect flight muscle stained with anti-Mhc antibody (red) and phalloidin (green) Scale bars are 200µm.

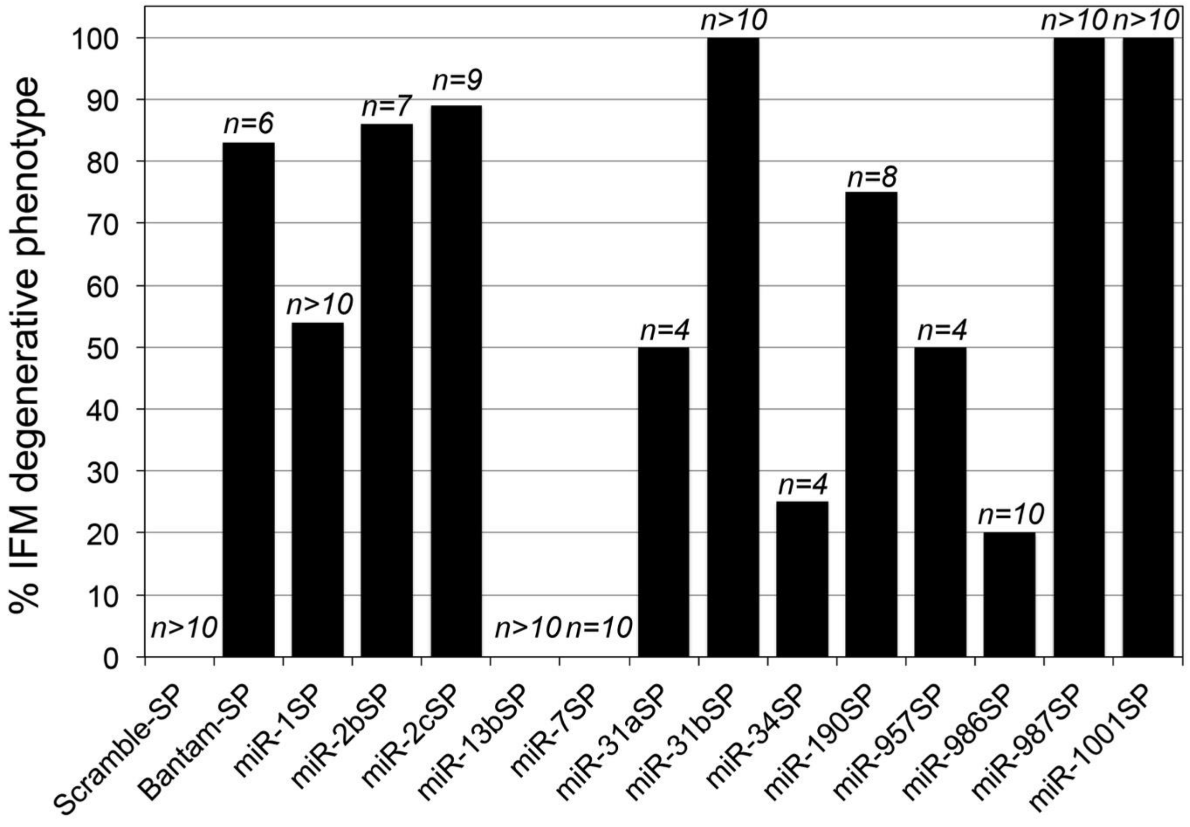

Supplementary Figure 4: Penetrance of IFM gross morphology phenotypes as assessed by anti-Mhc antibody and phalloidin staining of all positive hits from the primary flight screen. n = IFM Hemisegment.

dme-miRNA	Human ortholog	Functions	Methods	Models	References
miR-1	miR-1, 122, 206	Muscle development<br>Muscle adaptation<br>Muscle diseases	Overexpression<br>LOF<br>Profiling	*D. melanogaster*<br>*C. elegans*<br>*Homo sapiens*<br>*Mus musculus*<br>*Danio rerio*<br>*Cell culture*	Reviewed in:<br>Sokol, 2012<br>Kirby& McCarthy, 2013<br>Wang, 2013
K-box-miR (miR-2b, 2c, 13b)	miR-23	Muscular atrophy/hypertrophy	Overexpression<br>Profiling	*Homo sapiens*<br>*Mus musculus*<br>*Danio rerio*<br>*Cell culture*	Reviewed in:<br>Wang, 2013
miR-7	miR-7	Muscle diseases (DM1)	Profiling	*Homo sapiens*<br>*Mus musculus*	Fernandez-Costa et al., 2013
miR-31a, 31b	miR-31	Muscle development<br>Muscle adaptation<br>Muscle diseases (DMD)<br>Aging	Overexpression<br>LOF<br>Profiling	*Homo sapiens*<br>*Mus musculus*<br>*Stem cells*	Crist et al., 2012<br>Greco et al., 2009<br>Cacchiarelli et al., 2011<br>Roberts et al., 2012<br>Dmitriev, 2013<br>Russel et al., 2013<br>Hamrick et al., 2010
miR-34	miR-23a, 34b*, 34c-5p, 449a, 449b	Aging<br>Cell Death<br>Muscle diseases (DMD, myotonic dystrophy type-2)	LOF<br>Profiling	*D. melanogaster*<br>*Mus musculus*<br>*Homo sapiens*	Greco et al., 2009<br>Roberts et al., 2012<br>Greco et al., 2012<br>Boon et al., 2013

Supplementary Table 1: miRNAs implicated in muscle function from our screen that have orthologs shown to have functions in muscle through overexpression, loss of function or profiling experiments in various model organisms.

Supplementary References

1. Sokol, N. S. The Role of MicroRNAs in Muscle Development. *Curr. Top. Dev. Biol.* (2012). doi:10.1016/B978-0-12-387038-4.00003-3

2. Kirby, T. J. & McCarthy, J. J. MicroRNAs in skeletal muscle biology and exercise adaptation. *Free Radic. Biol. Med.* (2013). doi:10.1016/j.freeradbiomed.2013.07.004

3. Wang, X. H. MicroRNA in myogenesis and muscle atrophy. doi:10.1097/MCO.0b013e32835f81b9

4. Fernandez-Costa, J. M. *et al.* Expanded CTG repeats trigger miRNA alterations in Drosophila that are conserved in myotonic dystrophy type 1 patients. *Hum. Mol. Genet.* (2013). doi:10.1093/hmg/dds478

5. Crist, C. G., Montarras, D. & Buckingham, M. Muscle satellite cells are primed for myogenesis but maintain quiescence with sequestration of Myf5 mRNA targeted by microRNA-31 in mRNP granules. *Cell Stem Cell* (2012). doi:10.1016/j.stem.2012.03.011

6. Greco, S. *et al.* Common micro-RNA signature in skeletal muscle damage and regeneration induced by Duchenne muscular dystrophy and acute ischemia. *FASEB J.* (2009). doi:10.1096/fj.08-128579

7. Cacchiarelli, D. *et al.* miR-31 modulates dystrophin expression: new implications for Duchenne muscular dystrophy therapy. *EMBO Rep.* (2011). doi:10.1038/embor.2010.208

8. Roberts, T. C. *et al.* Expression Analysis in Multiple Muscle Groups and Serum Reveals Complexity in the MicroRNA Transcriptome of the mdx Mouse with Implications for Therapy. *Mol. Ther. — Nucleic Acids* (2012). doi:10.1038/mtna.2012.26

9. Dmitriev, P. *et al.* Simultaneous miRNA and mRNA transcriptome profiling of human myoblasts reveals a novel set of myogenic differentiation-associated miRNAs and their target genes.

10. Russell, A. P. *et al.* Regulation of miRNAs in human skeletal muscle following acute endurance exercise and short-term endurance training. *J Physiol* **000,** 1–17 (2013).

11. Hamrick, M. W. *et al.* The adipokine leptin increases skeletal muscle mass and significantly alters skeletal muscle miRNA expression profile in aged mice. *Biochem. Biophys. Res. Commun.* (2010). doi:10.1016/j.bbrc.2010.08.079

12. Greco, S. *et al.* Deregulated microRNAs in myotonic dystrophy type 2. *PLoS One* (2012). doi:10.1371/journal.pone.0039732

13. Boon, R. A. *et al.* MicroRNA-34a regulates cardiac ageing and function. doi:10.1038/nature11919
